# Self-Face Recognition in Schizophrenia: An Eye-Tracking Study

**DOI:** 10.3389/fnhum.2016.00003

**Published:** 2016-02-10

**Authors:** Catherine Bortolon, Delphine Capdevielle, Robin N. Salesse, Stéphane Raffard

**Affiliations:** ^1^Epsylon Laboratory, EA 4556Montpellier, France; ^2^University Department of Adult Psychiatry, CHU MontpellierMontpellier, France; ^3^Institut National de la Santé et de la Recherche Médicale U1061Montpellier, France; ^4^Movement to Health Laboratory, EuroMov, University of MontpellierFrance

**Keywords:** schizophrenia disorder, face recognition, self-face recognition, eye-tracking, eye movements

## Abstract

Self-face recognition has been shown to be impaired in schizophrenia (SZ), according to studies using behavioral tasks implicating cognitive demands. Here, we employed an eye-tracking methodology, which is a relevant tool to understand impairments in self-face recognition deficits in SZ because it provides a natural, continuous and online record of face processing. Moreover, it allows collecting the most relevant and informative features each individual looks at during the self-face recognition. These advantages are especially relevant considering the fundamental role played by the patterns of visual exploration on face processing. Thus, this paper aims to investigate self-face recognition deficits in SZ using eye-tracking methodology. Visual scan paths were monitored in 20 patients with SZ and 20 healthy controls. Self, famous, and unknown faces were morphed in steps of 20%. Location, number, and duration of fixations on relevant areas were recorded with an eye-tracking system. Participants performed a passive exploration task (no specific instruction was provided), followed by an active decision making task (individuals were explicitly requested to recognize the different faces). Results showed that patients with SZ had fewer and longer fixations compared to controls. Nevertheless, both groups focused their attention on relevant facial features in a similar way. No significant difference was found between groups when participants were requested to recognize the faces (active task). In conclusion, using an eye tracking methodology and two tasks with low levels of cognitive demands, our results suggest that patients with SZ are able to: (1) explore faces and focus on relevant features of the face in a similar way as controls; and (2) recognize their own face.

## Introduction

The ability to conceptualize and distinguish the self from others is a hallmark of human species (Gallup, 1977). Just after birth, infants already demonstrate a sense of their own body as a separate entity. Nevertheless, it is only later, around the age of 18 months, that they develop the ability to become the object of one’s own attention as someone separate from others, that is, an explicit self-awareness (Gallup, 1977; Rochat, 2003). Self-face recognition has been suggested to be an important indicator of explicit self-awareness. Thus, it has an important place in development and the understanding of the sense of conceptual “self” (Rochat and Striano, 2002).

Self-face recognition has been shown to be impaired in a variety of neurological or developmental disorders such as autism (Uddin et al., 2008), acquired brain injury, and Alzheimer disease (Adduri and Marotta, 2009). Concerning schizophrenia (SZ), studies on self-face recognition have provided contradictory results, showing either a global face recognition deficit (Lee et al., 2007; Zhang et al., 2012; Heinisch et al., 2013) or a specific self-face recognition deficit (Irani et al., 2006; Kircher et al., 2007) associated with hallucinations (Kircher et al., 2007). Poor performance in self-face recognition tasks is vulnerable to multiple interpretations. The main knowledge on self-face recognition in SZ comes from studies using paradigms that have attentional (Lee et al., 2007), memory (Kircher et al., 2007) and speed processing (Heinisch et al., 2013) demands, three cognitive functions impaired in patients with SZ (Keefe and Harvey, 2012) and impacting face processing (Bortolon et al., 2015). Studies applying other behavioral measures, in addition to an experimental design with low cognitive demands, could provide further insight into self-face recognition in patients with SZ. For instance, eye-tracking methodologies allow better understanding whether self-face recognition deficits originate from perceptual low-level stages (visual exploration) or from higher-level cognitive stages. Evidence showing that visual exploration of visual scenes is impaired in SZ (Beedie et al., 2011) suggests that low-level stages of visual processing can be also implicated in self face processing, which remains, however, to be explored.

Eye-tracking methodologies are known to provide a natural, continuous and online record of face processing, and more precisely indicate which features of the face are relevant and informative to each individual during self-face recognition (Duchowski, 2002; Rayner, 2009). For instance, it is well demonstrated that healthy individuals tend to fixate mostly the relevant features of the face, such as the eyes (Walker-Smith et al., 1977). Patients with SZ, on the other hand, present a restricted scan path strategy characterized by fewer and longer fixations, reduced saccades and avoidance of the relevant features of the face (Williams et al., 1999; Loughland et al., 2002). Interestingly, a recent study (Delerue et al., 2010) demonstrated that patients with SZ are capable of directing their attention to the relevant information of the face when they are directly requested to recognize a person (active condition) compared to when no specific instruction is provided (passive condition). Thus, they are capable of modulating their attention according to the task demands and explore different faces similarly to healthy controls in a quantitative and qualitative way.

Therefore, this study aimed to explore face scan path patterns in patients with SZ and healthy controls while looking at their own face, as well as a famous and an unfamiliar face under two different conditions: a passive and an active task (Delerue et al., 2010). Some important factors must be taken into account when evaluating self-face processing in patients suffering from SZ disorder. The first factor is whether patients suffering from SZ disorder present a deficit in processing faces in general, familiar faces or specifically their own face. For that reason we included in our experiment an unknown face and a famous face that they have previously seen and were able to correctly recognize. A second factor that must be considered is the habituation with the image, which might impact face exploration and also face recognition. Thus, the morphing procedure was applied in order to prevent habituation. Additionally, the morphing procedure enables the creation of a mismatch between the mental representation of the face, especially of one’s own face, and the external presentation, which may guarantee that participants will direct their attention to the different faces while exploring them and making the judgments (Kircher et al., 2000). This procedure also enables to better understand self-other boundaries in terms of visual perception in patients suffering from SZ. By showing faces that are similar to the self, but are not the same, we can observe whether patients with SZ tend, as healthy controls (Uddin et al., 2006), to recognize those faces as more similar to their own face or instead more similar to someone’s else face.

Previous researchers (Darke et al., 2013; Bortolon et al., 2015) argued that cognitive deficits could impact face processing depending on the experimental design of the task. Moreover, Delerue et al. (2010) suggested that when explicit instructions were provided to the participants during a simple face processing task with no time constraints, patients with SZ were able to direct their attention to the relevant features of the face like healthy controls. Based on these researches, the present study aims to explore whether this pattern of response can be observed in SZ disorder during self-face processing. If abnormal scan path patterns are associated with self-face recognition deficits, it would suggest that attentional/perceptual deficits impact the recognition of one’s own face in SZ. Conversely, if self-face recognition deficits are not associated with abnormal scan path patterns, it would indicate a specific impaired ability to recognize one’s own face, since the present task imposes no memory or speed processing demands.

## Materials and Methods

### Participants

Before the experiment, we calculated the sample size based on the previous study published by Delerue et al. (2010). We used the interaction between different factors in order to choose an effect size. Among the factors, we took into account in this calculation our main hypothesis, that is, patients with SZ would present impaired face exploration only during the passive task, but not during the active task. In other words, the interaction between groups (schizophrenia vs. healthy) and tasks (passive vs. active; two measurements) would be significant. Another factor we considered was the interaction between task and face (six measurements). We calculated the sample size with a power set at 90%, α risk at 5%, and an effect size of that varied between 0.64 and 0.68 (Cohen’s d). The software G*Power 3.1.9.2 was used. The calculations revealed that the highest sample size required would be composed by a total of 46 participants (23 participants in both groups).

Twenty five patients aged between 18 and 60 years meeting DSM-IV criteria for SZ, currently receiving inpatient or outpatient care were recruited in the Montpellier University Psychiatric Hospital. Diagnoses were made by a fully trained psychiatrist (D.C.) using the structured clinical interview for DSM–IV (SCID) (First et al., 1996). None of the patients were experiencing acute symptoms exacerbations at the moment of the inclusion. All patients received anti-psychotic medication. Exclusion criteria were: substance abuse, co-morbid neurological disorder, history of severe brain trauma or current electro-convulsive therapy.

Moreover, 25 healthy subjects matched on age, sex, and education level with patients with SZ were also included and screened for current psychiatric illness using the Mini-international Neuropsychiatric interview (Sheehan et al., 1998). They were excluded if they met criteria for any current axis I disorder of the DSM-IV-TR.

Five SZ patients and five healthy controls were excluded mostly due to technical problems [power outage, loss of the eye tracking signal, shorter gaze record – less than 7 s in more than 30% of the trials; (Roux et al., 2015)] and also because two patients could not keep the eyes opened during the experiment.

All participants needed to speak, read, and write French fluently and received a 40-Euro compensation to participate in the study. Moreover, all participants provided written informed consent prior to the experiment, approved by the National Ethics Committee (CPP Sud Méditérannée III, Nîmes, France, #2013.03.05ter and ID-RCB-2013-A00287-38) conforming to the Declaration of Helsinki.

### Materials

#### Clinical Measures

##### Medication

Mean chlorpromazine equivalent doses were computed.

##### Positive and Negative Syndrome Scale (PANSS) (Kay et al., 1987)

The PANSS is a 30-item rating scale completed by clinically trained research staff at the conclusion of a chart review and of a semi-structured interview, to assess symptom severity of patients with SZ.

#### Stimuli

Photographs of unknown people with neutral expressions were chosen from the NimStim Face Stimulus Set (www.macbrain.org) (Tottenham et al., 2009). Photographs of well-known famous people were taken from the internet (famous faces; e.g., Tom Cruise). We used only famous faces that participants were able to correctly identify and label. Frontal view pictures of each participant’s face with a neutral expression were taken the day before the experiment using an 8-megapixel digital camera (Canon PowerShot SX40). Photographs were ethnicity and gender-matched.

All pictures were edited in the Adobe Photoshop to match pictures for luminance, to crop each photograph into an oval encircling the eyes, the nose, and mouth, removing visual cues about hair and clothing, and resized using a scale based upon a resolution of 200 pixels. Stimulus size was 598 × 900 pixels (13.7° × 20.8°). Self-faces were mirror-reversed.

**FIGURE 1:**
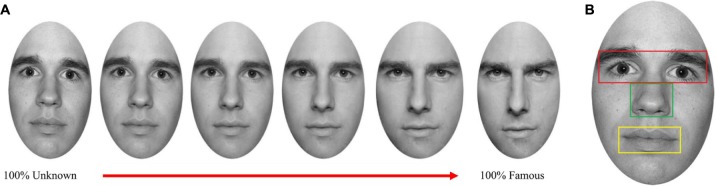
**(A)** Examples of the morphing continuum between 100% Unknown face and 100% Famous face. **(B)** Example of the relevant features of the face analyzed.

Participants’ faces were morphed with both an unknown and a famous face using Fantamorph software (Abrosoft V.4). Moreover, the famous face was morphed with the unknown face (**Figure 1A**). These three morphing procedures resulted in 12 unique faces, each morphed to a varying extent (containing 60, 80, 100% of the self or 60, 80, 100% of the famous/unknown face). Thus, 15 images were obtained containing 12 morphed pictures and three original pictures of the participant, famous and unknown faces.

The experimental task was designed and presented using E-prime software. All instructions and images were displayed on a white background in the center of the monitor, with a resolution of 1920 × 1080 pixels.

#### Eye Tracker Device

Applied Science Laboratory Mobile Eye XG eye-tracker consists of a head-mounted system built into a pair of glasses and a small, portable recording device. A scene camera, coinciding with the participant’s line of sight, recorded the scene in front of the person with a field of view of about 50° (horizontal) by 40° (vertical). Pupil and corneal reflections were recorded monocularly from the video image of the right eye at 30 Hz. Point of regard was then superimposed over the scene image as a circular cursor, allowing measurement of what was being looked at in each frame of the recorded video. Moreover, the mobile eye trackers have an instrumental spatial resolution of approximately 0.1°, and yield typical gaze position accuracy of 0.5–1°.

With the records that were obtained from the Applied Science Laboratory Mobile Eye XG eye-tracker, both temporal (duration) and count (number of fixations) were analyzed. A fixation was defined as a set of consecutive gaze coordinates, confined within a 1° of visual field for duration of 100 ms or more. The first fixation of each trial was excluded since it fell always in the center of the face around the nose region.

The spatial variables analyzed were the relevant features of the face (eyes, nose, and mouth), and irrelevant areas. The features of the face were defined as boxes around the respective facial features (**Figure 1B**) similar to Stacey et al. (2005). The boxes fitted the size of the facial features of each participant.

A “feature index” was calculated by subtracting the number of fixations on relevant features of the face by the number of fixations on irrelevant areas, and then divided by the total number of fixations. A negative value indicates a greater number of fixations on irrelevant areas, while a positive number indicates a greater number of fixations on the relevant features of the face. Eye tracking data were analyzed using Applied Science Lab (ASL) Results.

### Procedure

The first session was devoted to taking the participant’s photographs and evaluating patients’ symptomatology (PANSS). Then, we confirmed with the participants whether s/he was able to recognize the famous person.

The second session was intended to perform the face-recognition task. Participants were tested individually in a 20-min session whilst sitting. All participants positioned their head on a chin rest, with the eyes positioned centrally at a distance of 60 cm from the stimulus monitor.

At the beginning of each task, participants were presented with the instructions followed by a verbal explanation to ensure that they understood the instructions. A target display of nine dots covering the whole screen was used for calibration of eye position. After calibration, individuals always performed the passive task first, for which they were requested to look at the screen. Each face was displayed centrally during 7 s. A fixation cross was displayed during 1 s between each face during the passive task. This task was followed by the active task in which participants were requested to respond verbally whether the face presented was similar to themselves, to a famous person, or an unknown person. They were also instructed to provide a verbal answer only after the image disappeared. The investigator took note of all participants’ responses. The interval between each picture in the active task depended on the time participants took to respond. Moreover, a fixation cross was displayed during 1 s before each face. The same 15 pictures were shown in the passive and active viewing task, thus each face was presented twice. The order of face presentation was randomized for each participant.

### Data Analysis

#### Data Preparation

Skewed measures were transformed prior to analyses. Inversion transformations were applied to the duration of fixations.

#### Analysis

We compared groups’ characteristics using the Student *t*, Mann–Whitney *U* or χ^2^ tests. Repeated Measures ANOVA or a Student *t*-test was run on temporal and spatial variables with group (patient vs. control) as a between-subjects factor. Correlation analyses were performed to examine possible confounding effects of age, education, illness duration, and medication. SPSS (The Statistical Package for the Social Sciences version) 17.0 was used. Bonferroni *post hoc* analyses were carried out using Statistica 8. Graphics were constructed using Microsoft Excel.

## Results

### Social Demographic and Clinical Characteristics

Sociodemographic and clinical characteristics of the participants are presented in **Table 1**.

**Table 1:** Sociodemographic and clinical characteristics of the sample.

	Schizophrenia patients (*N* = 20)		Healthy controls (*N* = 20)		Statistics
			
	*M*	*SD*	*M*	*SD*	
Age	32.25	9.45	30.15	5.21	*t*(29.6) = 0.870, *p* = 0.390, *d* = 0.275, 95%CI[-2.78 – 6.98]
Education (years)	11.05	2.48	11.21	1.08	*U* = 152.50, *p* = 0.296, *r* = 0.245
Gender					χ^2^ = 0.229, *p* = 0.633
*Male n/%*	17	85	18	90	
PANSS					
*Positive symptoms*	8.65	1.93			
*Negative symptoms*	14.80	6.96			
*General psychopathology*	26.90	5.86			
Medication (chlorpromazine equivalents)	771.75	468.75			
Illness duration (years)	6.41	6.19			


### Eye-Tracking Analysis

#### Number of Fixations

**FIGURE 2:**
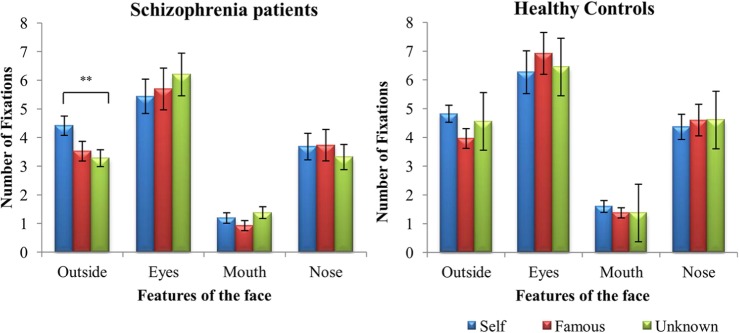
**Mean (±SD) difference values representing number of fixations for schizophrenia patients and healthy controls: face vs. features.** ^∗∗^*p* < 0.01.

Initially faces were grouped under the labels of self, famous and unknown when they contained more than 50% of the identity in accordance with (Uddin et al., 2005). For example, images morphed containing >50% of self-face would be designated as “self.” A 2 (groups) × 2 (tasks) × 3 (faces) × 4 (features of the face) mixed ANOVA was performed on the number of fixations, considering that faces were grouped accordingly (**Figure 2**). Results revealed a main effect of group, *F*(1,38) = 5.840, *p* = 0.021, $\eta_{p}^{2}$ = 0.133, task, *F*(1,38) = 15.365, *p* = 0.0001, η^2^ = 0.288, and feature, *F*(3,114) = 37.946, *p* = 0.0001, $\eta_{p}^{2}$ = 0.500. Overall, patients with SZ explored the different faces less than controls. However, Bonferroni *post hoc* analyses showed that both groups looked more at faces during the active task than during the passive one (*p* < 0.03). Moreover, they focused more on the eyes than on the other features of the face (*p* < 0.001), and less on the mouth than outside the relevant features of the face (*p* < 0.001), and the nose (*p* < 0.001) during face exploration.

Furthermore, four interaction effects were found: task and features of the face, *F*(1.923,73.073) = 3.812, *p* = 0.028, $\eta_{p}^{2}$ = 0.091, task and face, *F*(1,76) = 5.263, *p* = 0.007, $\eta_{p}^{2}$ = 0.122, feature and face, *F*(4.755,180.687) = 5.829, *p* = 0.0001, $\eta_{p}^{2}$ = 0.133, and between features of the face, face and group, *F*(6,228) = 3.017, *p* = 0.007, $\eta_{p}^{2}$ = 0.074. *Mauchly’s* test indicated that the assumption of sphericity had been violated for the interaction between task and feature, χ^2^(5) = 37.967, *p* = 0.0001, and for the interaction between feature and face, χ^2^(20) = 34.846, *p* = 0.001, therefore degrees of freedom were corrected using Greenhouse–Geisser estimates of sphericity for interaction between task and feature (ε = 0.641) and for the interaction between feature and face (ε = 0.792). Bonferroni *post hoc* analyses showed that both groups spent more time looking outside the relevant features during the active than the passive task (*p* = 0.03).

During face exploration, patients with SZ looked more outside the relevant features of the self-face compared to the other two faces (*p* = 0.005). Healthy controls explored the three faces quantitatively in a similar way (*p* > 0.05). Finally, no significant difference was found between patients with SZ and healthy controls on the number of fixations on the eyes, nose, and mouth for each of the three faces.

Regarding the second set of analysis, a 2(groups) × 2(tasks) × 4(features of the face) × 5(levels of morphing) ANOVA was performed for each identity: self, famous and unknown. The details of all results can be found in the Supplementary File. Analysis of self-face morphing continuum revealed a main effect of group, *F*(1,37) = 6.698, *p* = 0.01, $\eta_{p}^{2}$ = 0.15, task, *F*(1,37) = 4.467, *p* = 0.04, $\eta_{p}^{2}$ = 0.11, and feature, *F*(3,111) = 35.210, *p* < 0.0001, $\eta_{p}^{2}$ = 0.48, in addition to an interaction between task and morphing, *F*(4,148) = 2.594, *p* = 0.03, $\eta_{p}^{2}$ = 0.06. Bonferroni *post hoc* analysis revealed that differences between passive and active task are specific to the face containing 60% self and 40% unknown face. Regarding the famous face, besides the significant effect of group, *F*(1,37) = 8.257, *p* = 0.007, $\eta_{p}^{2}$ = 0.18, task, *F*(1,37) = 15.000, *p* < 0.0001, $\eta_{p}^{2}$ = 0.28, and feature, *F*(3,111) = 34.048, *p* < 0.0001, $\eta_{p}^{2}$ = 0.47, no other significant effect or interaction was found. A similar pattern was found for unknown faces. Only the main effect of group, *F*(1,37) = 10.510, *p* = 0.003, $\eta_{p}^{2}$ = 0.22, task, *F*(1,37) = 14.851, *p* < 0.0001, $\eta_{p}^{2}$ = 0.28, and feature, *F*(3,111) = 40.565, *p* < 0.0001, $\eta_{p}^{2}$ = 0.52, were observed. In both analyses, Bonferroni *post hoc* analysis revealed that: (1) healthy controls exhibited a larger number of fixations while exploring each face compared to patients with SZ; (2) both groups exhibited a larger number of fixations during the active than during the passive task; and (3) both groups fixated less the mouth than the other features of the face and more the eyes than outside. They also fixated more the eyes than the nose (*p* = 0.02), but for the famous continuum differences only approached significance (*p* = 0.07).

#### Feature Index for Number of Fixations

The differences in scanning behavior for each different face were examined using a 2 (groups) × 2 (tasks) × 3 (faces) repeated measures ANOVA. A significant effect of task, *F*(1,38) = 6.344, *p* = 0.016, $\eta_{p}^{2}$ = 0.143, and of face, *F*(2,76) = 8.043, *p* = 0.001, $\eta_{p}^{2}$ = 0.175, was found on the “feature index.” No significant effect of group was found, *F*(2,38) = 0.042, *p* = 0.839, $\eta_{p}^{2}$ = 0.001. Compared to self-face, participants presented a higher “feature index” for famous (*p* = 0.003) and unknown face (*p* = 0.023), indicating that when looking at these two faces participants paid more attention to the relevant features of the face than to the irrelevant ones. Moreover, both groups presented a higher “feature index” during the passive task than the active one.

Subsequently, a 2 (groups) × 2 (tasks) × 5 (morphing level) repeated measures ANOVA was performed for each identity morphing continuum. No significant effect was observed. A trend toward significance was observed for task on the unknown morphing, *F*(1,37) = 3.887, *p* = 0.056, $\eta_{p}^{2}$ = 0.09, indicating that participants looked more at different points while exploring the faces during the passive than during the active task.

#### Duration of Fixations

Due to the number of missing cases we decided not to perform a 2(groups) × 2 (tasks) × 4 (features of the face) × 3 (faces) repeated measures ANOVA. It would result in only 10 patients with SZ and 18 healthy controls. Missing cases were due to the fact that participants do not look at specific feature when exploring some faces. If they do not look at the feature, no duration of fixation can be evaluated. For instance, if a participant looked at the eyes and nose during the exploration of a famous face during the passive task, we did not have data about fixation of the mouth and outside the relevant features of the face for this participant. Consequently, this case would be automatically excluded from the analysis. Therefore, we first performed a 2 (tasks) × 3 (faces) repeated measures ANOVA with a between group factor (schizophrenia vs. controls) and within each group separately. Performing two ANOVAs separately would allow first to compare groups with the whole sample and then analyze the effect of the features of the face within each group with the reduced number of participants. Moreover, we performed a 2 (groups) × 2 (tasks) × 5 (morphing level) ANOVA in order to analyze the effect of the different levels of morphing within each identity on duration of fixation

**FIGURE 3:**
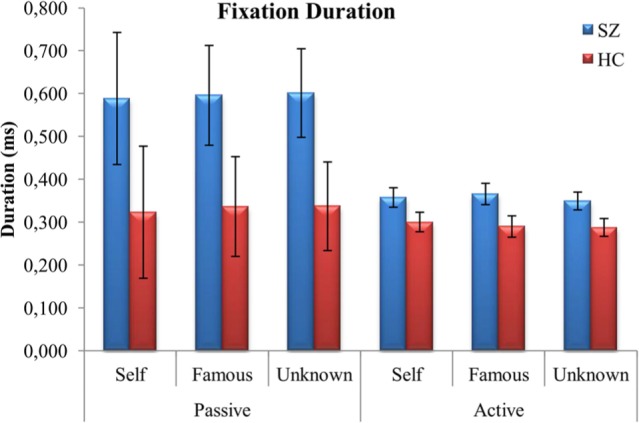
**Mean (±SD) values representing duration of fixations (ms) for both schizophrenia patients (SZ) and healthy controls (HC): face vs. task**.

Results first revealed a significant main effect of group, *F*(1,38) = 5.265, *p* = 0.027, $\eta_{p}^{2}$ = 0.122, and task, *F*(1,38) = 22.900, *p* < 0.001, $\eta_{p}^{2}$ = 0.376. Patients with SZ fixated longer than healthy controls, but both groups fixated longer during the passive task than during the active one. Moreover, an interaction between face and task was also found, *F*(1,38) = 4.898, *p* = 0.01, $\eta_{p}^{2}$ = 0.114. However, the results indicated that participants fixated longer during the passive than during the active task for all three faces (**Figure 3**).

Repeated measures ANOVA for patients with SZ revealed a main effect of feature, *F*(3,27) = 4.496, *p* = 0.011, $\eta_{p}^{2}$ = 0.333. Nevertheless, *post hoc* Bonferroni analyses revealed no significant differences between features. For healthy controls, results also revealed a main effect of feature, *F*(3,51) = 7.101, *p* < 0.001, $\eta_{p}^{2}$ = 0.295. Healthy controls fixated longer at the eyes than outside the relevant features.

When the effect of morphing level within each identity was analyzed, results revealed a significant effect of group only for the faces containing more than 60% of famous, *F*(1,37) = 4.569, *p* = 0.03, $\eta_{p}^{2}$ = 0.11, and unknown face, *F*(1,37) = 5.188, *p* = 0.03, $\eta_{p}^{2}$ = 0.12. An interaction between task and morphing level was also observed, *F*(4,148) = 2.812, *p* = 0.03, $\eta_{p}^{2}$ = 0.07. Differences between passive and active task in terms of fixation duration are limited to two faces: 80% unknown – 20% self (*p* = 0.0003) and 60% unknown – 40% famous (*p* = 0.03).

### Self-Face Recognition

**FIGURE 4:**
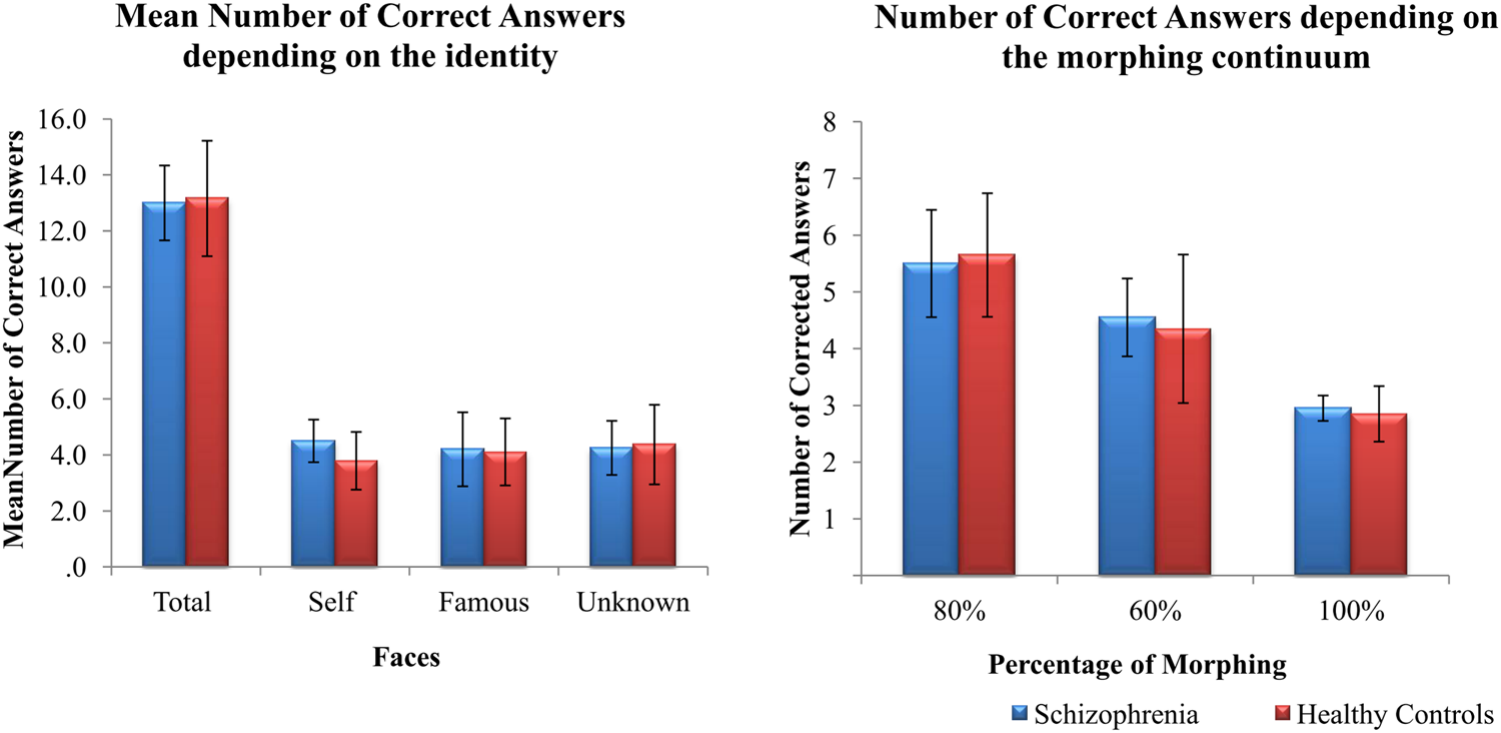
**Mean (±SD) values of correct answers during the active task **(A)** depending on the identity and **(B)** depending on the morphing continuum for both patients with schizophrenia and healthy controls**.

The number of correct answers reported during the active task was analyzed using a 2(groups) × 3 (faces) repeated measures ANOVA. No significant effects were found for group, *F*(1,37) = 0.764, *p* = 0.388, $\eta_{p}^{2}$ = 0.020, or face, *F*(2,74) = 0.219, *p* = 0.804, $\eta_{p}^{2}$ = 0.006 (**Figure 4**). We also analyzed whether the ambiguity of the images could impact on face performance. To this aim, we summed up the number of correct answers for faces containing 80, 60, or 100% of a face regardless of the identity (**Figure 4**). No significant differences were found between groups: 80%, *U* = 155, *p* = 0.336; 60%, *U* = 186, *p* = 923; 100%, *U* = 189.5, *p* = 989.

### Correlations Analysis

Bonferroni corrections were applied due to the high number of correlations performed (*p* < 0.001). After the correction no significant correlations were found between eye tracker measures and illness duration, clinical symptoms and medication dose. No significant correlations (*p* > 0.001) were found between accuracy and both eye tracker and clinical symptoms (**Table 2**).

**Table 2:** Correlation between clinical variables, eye tracking measures and number of correct answers during the face recognition task (active task) in patients with schizophrenia disorder.

		PANSS			Illness duration	Medication dose	Number of correct answers
					
		Positive symptoms	Negative symptoms	General psychopathology			
**Passive task**							
Average index	Correlation coefficient	-0.202	-0.163	-0.115	0.080	-0.114	
	*p-value*	0.394	0.493	0.629	0.780	0.633	
Number of fixations	Correlation coefficient	0.124	-0.278	0.291	0.079	-0.062	
	*p-value*	0.602	0.236	0.213	0.763	0.795	
Duration of fixations	Correlation coefficient	-0.075	0.353	-0.239	-0.038	0.175	
	*p-value*	0.753	0.127	0.310	0.884	0.460	
**Active task**							
Average index	Correlation coefficient	-0.096	-0.542	-0.073	-0.025	-0.147	-0.034
	*p-value*	0.688	0.014	0.761	0.925	0.554	
Number of fixations	Correlation coefficient	0.039	-0.216	0.340	-0.025	-0.029	-0.052
	*p-value*	0.871	0.360	0.140	0.925	0.904	
Duration of fixations	Correlation coefficient	-0.127	0.184	-0.327	0.067	0.005	0.032
	*p-value*	0.593	0.437	0.159	0.800	0.982	
Number of correct answers	Correlation coefficient	0.093	-0.278	0.088	0.011	-0.128	
	*p-value*	0.697	0.235	0.712	0.591	0.591	


## Discussion

Previous studies on self-face recognition in patients with SZ have provided contradictory results. Poor performance in tasks investigating face recognition could be attributed to different factors. Thus, it is difficult to establish whether previous reported impairments in recognizing one’s own face are due to a specific self-face recognition deficit or attributed to other confounding factors, such as attention and perceptual deficits (Bortolon et al., 2015). Therefore, we proposed to evaluate self-face recognition in patients with SZ using an eye-tracking methodology. Eye-tracking methodology helps better understand whether these attentional/perceptual deficits impact on self-face processing (Duchowski, 2002). Overall, our results showed that patients with SZ showed fewer and longer fixations during self-face processing compared to healthy controls, but the pattern of face exploration did not differ between them.

Previous studies using eye-tracking methodology in patients with SZ showed fewer and longer fixations during other’s face processing compared to healthy controls (Williams et al., 1999; Loughland et al., 2002). This result was confirmed in the present study, regardless of the identity of face (i.e., self, famous, and unknown), and type of tasks (i.e., passive, active). Furthermore, previous studies showed that identity did not impact the number of fixations inside and outside the relevant features of the face during a face recognition task (Stacey et al., 2005; Kita et al., 2010). Our results also confirmed this result for both groups, who presented a similar pattern of face exploration (feature index) regardless of the face identity or the type of task. In other words, both healthy controls and patients suffering from SZ explore the relevant features of the different faces in a similar way. Nevertheless, it does not corroborate a previous study (Delerue et al., 2010) that did not find significant differences between groups in terms of temporal and spatial measures, but reported that patients differ from healthy controls during the passive task when the proportion of fixations inside and outside (feature index) relevant features was considered. It is possible that the stimuli themselves elicited this pattern of exploration. First, we presented a highly known face (the self-face), a famous, and an unknown face, while Delerue et al. (2010) presented faces that were mostly unknown. Second, self and famous faces were morphed with an unknown face. At some steps of the morphing, for example, the morphology of some features of the face relies between the identities, resulting in ambiguity, which might elicit the exploration of the different features to try to figure out which identity they are actually seeing. This pattern might reflect a strategy to resolve the ambiguity present in the morphed faces as previously shown by Barton et al. (2006) in healthy controls. Thus, our results suggest that, although patients with SZ present reduced but longer fixations compared to healthy controls when exploring their own face (and also the other faces), they are still capable of directing their attention and processing the different relevant features of their own face.

During the active task, participants were also requested to answer whether they thought the face displayed was more similar to their own face, the famous face, or the unknown face. Contrary to some previous studies (Irani et al., 2006; Kircher et al., 2007), our results showed that patients with SZ are capable of recognizing their own face, the famous face, and of determining whether the face was unknown. Aforementioned, we used a morphing procedure also to create a mismatch between the mental representation of one’s own face and the external presentation. Patients were able to recognize their own face (the famous and the unknown face as well) even when this mismatch was presented with no time constraint. Although some studies have suggested that patients suffering from SZ might have some trouble recognizing other individuals’ face (Sachs et al., 2004; Kircher et al., 2007; Chen et al., 2008), in particularly under laboratory settings, recent reviews suggested that these deficits might be better explained by other cognitive deficits, notably, attentional and speed processing deficits (Darke et al., 2013; Bortolon et al., 2015). This issue is also observed in studies investigating self-face processing in SZ disorder. Previous studies employed tasks in which the cognitive demands might impact on face recognition, such as memory, attention, and processing speed (Kircher et al., 2007; Lee et al., 2007; Heinisch et al., 2013). Our task, conversely, requires low levels of memory and speed processing (Goghari et al., 2011). Therefore, our experiment provides evidence suggesting a spared explicit sense of self in SZ. It is possible; however, that other levels of physical self might be impaired. For instance, Ferri et al. (2012) evaluated explicit and implicit body recognition in patients suffering from SZ. Their results provided evidence of more important self-other discrimination impairment. More importantly, however, they showed that unlike healthy controls, patients did not present a self-advantage effect during the implicit task. They suggested that SZ disorder might be characterized by disturbances of the implicit bodily self-awareness. Further studies should also evaluate implicit self-face recognition in patients with SZ disorder.

Moreover, self-face recognition might be disrupted under other circumstances. For instance, during mirror self-recognition, individuals need to be aware of their appearance and also of the equivalence between the visual information of the self’s movements in the mirror and the proprioceptive information provided from the same movement. A disruption in the multisensory integration that gives place to the sense of body ownership could be implicated in self-mirror recognition in patients with SZ. Therefore, studies investigating mirror self-recognition, in particularly, in daily life could provide further insight into the relationship between self-disturbances and self-recognition. Moreover, understanding self-recognition in everyday life could also provide more information regarding the feelings associated with their own image, such as feelings of strangeness (Bredart and Young, 2004).

This study has some limitations. As our sample was relatively small, it is difficult to generalize our results. It is possible that the relatively mild severity of psychopathology in our patient sample precluded significant differences between groups. Moreover, our sample is mostly composed of men, and previous studies have found sex differences in facial scanning (Rennels and Cummings, 2013). More studies, with larger and more heterogeneous samples would provide further insight into the questions investigated in this study. Finally, we used the same pictures in the passive and active task. Thus, it is possible that the participants’ acquaintance with the face might affect our results. Further studies should take into account this limitation by using different self, different unknown and different famous faces in each task.

An important detail of the present results is the fact we could not consider all participants when analyzing the duration of fixation, in particularly patients with SZ. Since several patients avoided looking at some parts of some faces during the tasks, it was impossible to obtain the duration of the fixation. Thus, these participants were automatically excluded from the analysis of variance when considering the duration of fixation within each feature of the face. This pattern was observed in 10 patients and only in two healthy controls. Thus, the analysis of both fixations and duration of fixations specifically for each facial feature were not comparable since they were not based on the same number of participants. Although it might be considered as a limitation of the present analysis, it might also reflect a specific pattern of face exploration in SZ patients characterized by the avoidance of some facial features, which should be further investigated in a future study. An interesting method would be to employ the iMap. The iMap is a method for statistical fixation mapping of eye movement data developed by Caldara and Miellet (2011) that does not require segmentation of the experimental images into areas of interested. Thus, it enables to compare the fixation maps between groups.

In summary, our results first suggested that, although patients with SZ presented fewer and longer fixations, they are capable of paying attention to the relevant features of their own face similarly to healthy controls, regardless of the task instruction. Moreover, our results showed that patients with SZ are capable of correctly recognizing and labeling their own face as being theirs, when memory and speed processing demands of the task are low. As regards the attention and perceptual aspects, our results suggested that the way patients with SZ explore their own face does not impact on their ability to recognize it. Therefore, it seems that this dimension of the self is spared in patients with SZ.

## Author Contributions

CB, DC, SR, and RS contributed to the study design. DC and CB recruited and assessed the patients. CB performed the statistical analysis and wrote the first draft. CB, SR, and RNS prepared the final manuscript, with feedback from the other authors.

## Conflict of Interest Statement

The authors declare that the research was conducted in the absence of any commercial or financial relationships that could be construed as a potential conflict of interest.
