# Supplementary material for: Self-Face Recognition in Schizophrenia: An Eye-Tracking Study
Source: Front Hum Neurosci. 2016 Feb 10;10:3. doi: 10.3389/fnhum.2016.00003 (PMC4748053; doi:10.3389/fnhum.2016.00003)
Supplement: Supplementary file 1 [file Table_1.DOCX]

**Supplementary Data**

| Table 1. Statistical Results of the Repeated Measures ANOVA for the Self Morphing | | | |
| --- | --- | --- | --- |
| Source | F | Sig. | Partial Eta Squared |
| Morphing Level | .817 | .516 | .022 |
| Morphing Level × Group | .893 | .470 | .024 |
| Feature × Morphing Level | .844 | .605 | .022 |
| Feature × Morphing Level × Group | .990 | .457 | .026 |
| Feature × Group | .292 | .831 | .008 |
| Task × Morphing Level × Group | 1.350 | .254 | .035 |
| Task × Feature | 1.119 | .345 | .029 |
| Task × Feature × Morphing Level | .468 | .933 | .012 |
| Task × Feature × Morphing Level × Group | 1.446 | .142 | .038 |
| Task × Feature × Group | 1.349 | .262 | .035 |
| Task × Group | .086 | .770 | .002 |

Table 2. Statistical Results of the Repeated Measures ANOVA for the Famous Morphing

|  | | | |
| --- | --- | --- | --- |
| Source | F | Sig. | Partial Eta Squared |
| Morphing level | .631 | .641 | .017 |
| Morphing level × Group | .553 | .697 | .015 |
| Feature × Morphing level | .875 | .573 | .023 |
| Feature × Morphing level × Group | 1.555 | .102 | .040 |
| Feature × Group | .373 | .773 | .010 |
| Task × Morphing level | 1.554 | .190 | .040 |
| Task × Morphing level × Group | .538 | .708 | .014 |
| Task × Feature | .942 | .423 | .025 |
| Task × Feature × Morphing level | .363 | .975 | .010 |
| Task × Feature × Morphing level × Group | 1.455 | .138 | .038 |
| Task × Feature × Group | 1.016 | .389 | .027 |
| Task × Group | 1.379 | .248 | .036 |

Table 3. Statistical Results of the Repeated Measures ANOVA for the Unknown Morphing

|  | | | |
| --- | --- | --- | --- |
| Source | F | Sig. | Partial Eta Squared |
| Morphing Level | .150 | .963 | .004 |
| Morphing Level × Group | .418 | .795 | .011 |
| Feature × Morphing Level | .606 | .838 | .016 |
| Feature × Morphing Level × Group | 1.341 | .192 | .035 |
| Feature × Group | .325 | .807 | .009 |
| Task × Morphing Level | .352 | .842 | .009 |
| Task × Morphing Level × Group | .906 | .462 | .024 |
| Task × Feature | .658 | .580 | .017 |
| Task × Feature × Morphing Level | .653 | .796 | .017 |
| Task × Feature × Morphing Level × Group | .391 | .967 | .010 |
| Task × Feature × Group | .736 | .533 | .020 |
| Task × Group | 2.813 | .102 | .071 |
